# Home mechanical ventilation: a narrative review and a proposal of practical approach

**DOI:** 10.1016/j.bjane.2025.844595

**Published:** 2025-01-26

**Authors:** Simone Chaves Fagondes, Carmem Lúcia Oliveira da Silva, Anneliese Hoffmann, Rita de Cássia Guedes de Azevedo Barbosa, Daiane Falkembach, Ângela Beatriz John

**Affiliations:** a Hospital de Clínicas de Porto Alegre, Serviço de Pneumologia, Programa de Residência Médica em Medicina do Sono e Suporte Ventilatório, Porto Alegre, RS, Brazil; b Hospital de Clínicas de Porto Alegre, Serviço de Pediatria, Porto Alegre, RS, Brazil; c Hospital de Clínicas de Porto Alegre, Serviço de Pediatria, Unidade de Pneumologia Infantil, Porto Alegre, RS, Brazil; d Programa VENTLAR do Hospital Infantil João Paulo II – Fundação Hospitalar do Estado de Minas Gerais, Belo Horizonte, MG, Brazil; e Hospital de Clínicas de Porto Alegre, Serviço de Fisioterapia, Porto Alegre, RS, Brazil

**Keywords:** Home care services, Noninvasive ventilation, Pediatrics, Respiration, artificial, Respiratory insufficiency, Ventilators, mechanical

## Abstract

Growing evidence of the benefits of home ventilatory support in patients with chronic respiratory failure along with technological advances in ventilators have enabled their use in overly complex situations, shaping a new scenario for physicians. This has further given rise to new challenges related to their incorporation into current medical practice. However, this evolution needs to be coupled with knowledge and skills of physicians who are willing to prescribe Home Mechanical Ventilation (HMV), in order to prevent them from making inappropriate choices or adjustments that may ultimately have ethical and legal implications. This article aims to provide guidance and information to support the indication for HMV and the ventilation modalities to be implemented, review basic ventilation concepts, including the ventilator modes most commonly used in patients outside the hospital setting, list the brands and models available in the Brazilian market, provide the means for obtaining equipment for HMV, and finally, describe the requirements for selection of equipment, taking into account the individual characteristics of the patient to ensure safe perioperative care and earlier dehospitalization.

## Introduction

Home ventilatory support has allowed the dehospitalization of patients with a wide range of medical conditions, who until recently occupied hospital beds for prolonged periods, especially in tertiary care hospitals. Among the ventilatory support options, Noninvasive Ventilation (NIV) has gained increasing acceptance in clinical practice.^1^ This ventilation modality is characterized by not requiring an invasive interface between patient and ventilator and has proven to be a safe method, easy to understand for the care team, more comfortable for the patient and with significant reduction in morbidity associated with conventional ventilation. The use of NIV is increasing, ranging from chronic conditions where it has a well-defined role supported by robust evidence to the setting of acute disease.^2^, ^3^, ^4^, ^5^, ^6^, ^7^, ^8^

This growing success is closely related to significant technological advances in ventilators, which have enabled their use in increasingly complex clinical situations.^9^^,^^10^ However, technological advances need to be coupled with knowledge for the correct indication and optimization of the resources available in the equipment, in addition to the development of skills and competences to solve the problems that may arise during therapy. For this reason, several countries have already implemented specialist centers with expertise in patients with difficult ventilator weaning and management of long-term ventilated individuals.^7^^,^^10^, ^11^, ^12^ In Brazil, centers with home ventilation programs are still rare and concentrated in a few regions of the country. One of the oldest and most structured programs is the VENTLAR program, implemented in 2002 by the Hospital Foundation of the State of Minas Gerais (FHEMIG), which provides domiciliary medical care to patients with Neuromuscular Disease (NMD).

Home Mechanical Ventilation (HMV) devices are medical equipment designed to assist and promote patient ventilation outside the hospital setting and should only be used under medical prescription and supervision with appropriate training and qualification for this purpose, since their inappropriate use may have legal implications for the patient's attending physician. Given the complexity of caring for a ventilator-dependent patient at home, it is also desirable that, in addition to the care provided by a multidisciplinary team that includes a physician, a nurse, and a physical therapist, the ventilator supplier be fully engaged in providing quick responses to the team's demands, especially towards clinical complications.^13^, ^14^, ^15^

Once HMV has been indicated, it is important to have an individual discharge plan for each patient, aiming to provide training to patients and their family members and caregivers about the operation of home ventilatory support devices and development of skills to identify and solve potential life-threatening events, minimizing the risks associated with the use of this technology.^14^, ^15^, ^16^, ^17^

## Methods

This narrative review introduces applicable points of HMV. We describe the indications for HMV, the main concepts used in HMV, the factors that influence successful delivery, the criteria for selection of equipment, and the minimum requirements for the implementation of HMV. We searched the PubMed (MEDLINE) database for papers written in English, Spanish, Portuguese, German, or French with no date restrictions. No age filter was applied. Our search strategy included terms such as “noninvasive ventilation”, “home mechanical ventilation”, “chronic respiratory failure”, “chronic respiratory insufficiency”, “alveolar hypoventilation”, “chronic hypoventilation”, “neuromuscular disease”, “COPD”, “thoracic restrictive disorders”, “obesity hypoventilation syndrome” and “caregiver”. Boolean operators “AND” and “OR” were used. The reference lists of selected articles were also screened for additional relevant papers.

### Indications for HMV

Evidence on the benefits of HMV to control chronic hypoventilation symptoms improving survival and reducing hospital admissions has expanded the range of clinical indications both in the adult and pediatric populations. Presently, patients with extreme prematurity, patients with NMD, especially with a rapid and/or progressive course, patients with Chronic Obstructive Pulmonary Disease (COPD), patients with thoracic restrictive disorders and obesity hypoventilation syndrome, and more recently, patients with extensive pulmonary sequelae of COVID-19 can benefit from home ventilatory support (Table 1).^8^^,^^13^^,^^15^^,^^17^, ^18^, ^19^, ^20^, ^21^

**Table 1:** Most common indications for Home Mechanical Ventilation.

Type of ventilatory dysfunction	Indications
Upper airway obstruction	Obstructive sleep apnea not controlled by Continuous Positive Airway Pressure (CPAP)
Dynamic lower airway obstruction	Tracheomalacia
	Bronchomalacia
Alveolar hypoventilation (chronic respiratory failure with hypercapnia)	- Neuromuscular diseases:
	a) Diseases that affect the spinal cord (e.g., trauma) and motor neuron diseases – such as post-polio syndrome, amyotrophic lateral sclerosis (ALS), and spinal muscular atrophy (SMA), among others
	b) Motor nerve diseases (e.g., Charcot-Marie-Tooth disease)
	c) Neuromuscular junction disorders (e.g., myasthenia gravis)
	d) Muscle diseases – such as muscular dystrophies (Duchenne and Becker's), limb-girdle dystrophy, myotonic dystrophy, myopathies, and genetic metabolic diseases (e.g., glycogen storage diseases), among others
	- Diaphragmatic paralysis
	- Obesity hypoventilation syndrome
	- Chest wall deformities such as kyphoscoliosis (idiopathic, secondary to other diseases, and congenital), ankylosing spondylitis, acquired chest wall abnormalities (after thoracoplasty, fibrothorax, and chest wall tumors), and abnormalities secondary to abdominal processes, including morbid obesity and ascites
	- Advanced chronic lung diseases of obstructive or restrictive pattern – such as chronic obstructive pulmonary disease (COPD), interstitial pulmonary involvement with or without fibrosis, cystic fibrosis, bronchiectasis, bronchiolitis obliterans, and apnea of prematurity, among others
	- Central alveolar hypoventilation syndromes – such as congenital central alveolar hypoventilation syndrome, Arnold-Chiari malformation, neurometabolic disorders, acquired causes such as brain tumors, encephalitis, cerebral infarction, and sequelae of traumatic brain injury or neurosurgery, among others

Evidence of the role of HMV in COPD patients with chronic hypercapnia has recently emerged. HMV in patients with COPD is the most studied setting and has demonstrated the most meaningful level of evidence to date. In patients with COPD, home Bilevel Positive Airway Pressure (BPAP) (compared with no device) was associated with decreased mortality, need for intubation, and hospital admissions. These individuals also had fewer hospital admissions with HMV than those with BPAP, Continuous Positive Airway Pressure (CPAP), or no device.^22^ The American Thoracic Society Clinical Practice Guideline suggests the use of nocturnal NIV in addition to usual care for patients with chronic stable hypercapnic COPD (conditional recommendation, moderate certainty).^23^

The American College of Chest Physicians Clinical Practice Guideline and Expert Panel Report recommend using NIV for treatment in patients with NMD and chronic respiratory failure (strong recommendation, very low certainty of evidence) and in those with NMD and sleep-related breathing disorders (conditional recommendation, very low certainty of evidence).^24^ In patients with NMD, home BPAP (compared with no device) was associated with lower mortality and better quality of life. In patients with obesity hypoventilation syndrome, home BPAP (compared with no device) was associated with lower mortality.^22^ Current evidence is not available to assess the comparative effectiveness of many device capabilities in patient outcomes.^22^

In some instances, it may be necessary to initiate HMV in hospital. A prospective, multicenter, non-inferiority trial comparing the effectiveness of adaptation to NIV performed in the ambulatory or hospital setting in patients with chronic respiratory failure secondary to restrictive thoracic disease, obesity hypoventilation syndrome, or NMD showed that adaptation to NIV in the ambulatory setting is not inferior to hospital adaptation in terms of therapeutic equivalence in stable patients. Outpatient adaptation may represent a cost saving for the health care system.^25^

The successful implementation of HMV will depend on clinical and psychosocial factors (Table 2).^26^

**Table 2:** Factors that influence HMV implementation and continuity.

Clinical factors	Psychosocial factors
Clinical indication for HMV (underlying disease)	Patient and family/caregiver motivation
Type of equipment and ventilatory mode	Level of anxiety and ability to accept and adapt to therapy
Adverse effects of HMV (alterations associated with interface such as unintentional and excessive leaks around the mask, skin lesions, and aerophagia)	Family support
Patient-ventilator asynchrony	Home conditions (presence of adequate and naturally ventilated room, reliable electricity supply, basic sanitation, and running water)
Current stage of the disease	Fast connection technology available for prompt communication
Level of independence	Easy access to the referral medical center
Presence of comorbidities	Access to the primary health network suitable for care
Likelihood of disease progression	Home with difficult access for health team arrival
Level of dependence on ventilatory support	Intellectual disability that impairs treatment
Tracheostomy	High cost of equipment and consumables and scarce public health policies in this area

Source: Adapted from Piper AJ^26^ and Zampoli M.^41^

HMV can be related to a difficult life situation with psychological, social, and existential challenges. Although HMV increases the well-being of patients and facilitates a community- and home-based lifestyle compared with institutional-based treatment, some patients undergoing HMV describe experiences that this treatment causes distress, anxiety, vulnerability, fear, loneliness, and emotional responses such as hopelessness in their lives. A constant feeling of uncertainty and dependence on caregivers and equipment is also reported. High stress levels among patients have been reported in some studies. Difficulties in communication and the frustration with the possibility of loss of speech are described as a relevant negative impression of HMV users. Patient and family education, sharing information, resistance and resilience strategies, and music therapy may be helpful in reducing stress levels and improving patients’ quality of life.^27^, ^28^, ^29^

In addition, caregivers describe a sense of duty to take care of loved ones, but they also suffer a significant restriction of their own time with a negative impact on their physical and mental health. The initial transfer home is highlighted as the most stressful part of the process.^30^ Caregivers most frequently experience a medium level of burden, but female caregivers may experience a higher level of burden which invasive ventilation tends to increase. The financial situation seems to be inversely related to the burden. Interventions to ensure that caregivers overcome these responsibilities should be undertaken where some positive coping strategies may reduce the level of burden.^29^^,^^31^

### Main concepts used in the application of home ventilatory support^32^

#### IPAP

Inspiratory Positive Airway Pressure (IPAP) is the preset pressure in the ventilator that will be released during inspiration. The support pressure will then be established (IPAP – EPAP) to provide assistance for inspiration and reduce ventilatory effort.

#### EPAP

Expiratory Positive Airway Pressure (EPAP) is the preset pressure in the ventilator that will be maintained at the end of expiration. EPAP allows the maintenance of upper airway patency and helps recruit and maintain lung volume, improving oxygenation. It is also necessary to ensure sufficient expiratory flow to reduce CO_2_ from the ventilatory dead space, enabling the removal of CO_2_ from the ventilator circuit and preventing re-inhalation. Some devices allow the adjustment of an EPAP interval that adjusts to the level of Positive End-Expiratory Pressure (PEEP) applied in response to patient-related changes (auto EPAP).

#### Volume

The adjustment of the tidal volume or minute volume, in addition to the IPAP, is possible in some devices. In this mode, the device releases a fixed volume and a volume-adjusted inspiratory flow in each ventilation. The term pressure support with volume control, also known as VAPS (volume-assured pressure support), refers to a feature available in some devices that allows the establishment of the target level of ventilation through the variation of the support pressure provided by the ventilator.

#### Respiratory rate

A back-up respiratory rate can be adjusted to ensure a minimum level of ventilation for the patient. If the patient's spontaneous respiratory rate is lower than this level, the equipment will provide the necessary ventilation to ensure the preset minimum value. This option is available on some devices, in S/T ventilatory mode.

#### Inspiratory time

In certain ventilatory modes, it is possible to adjust the inspiratory time. This feature allows the equipment to release inspiratory pressure only for the adjusted period, regardless of the patient's spontaneous ventilatory pattern. Some devices allow the adjustment of minimum and maximum inspiratory times in order to avoid problems, such as premature or late cycling, reducing the likelihood of patient-ventilator asynchrony.

#### Triggering

The equipment's ability to detect changes in flow or pressure resulting from the onset of inspiration by the patient, triggering a change from expiratory to inspiratory pressure. Excessive leakage or airborne leakage can affect the firing of the equipment, resulting in self-triggering or even impairing the detection of the patient's inspiratory effort. Some devices allow the adjustment of the sensitivity of the shot.

#### Cycling

When the patient exhales, the equipment detects a change in airflow and activates its cycling, reducing expiratory pressure. Some devices allow the adjustment of the sensitivity of the cycling, which can occur by flow or time.

#### Rise time

This is the time needed to reach IPAP after the beginning of the inspiratory phase.

#### Fall time

This is the time needed for the IPAP to drop after the equipment starts expiring.

### Main ventilator modes used in home ventilation devices^1^^,^^19^

Spontaneous modes (S or E)

Ventilation occurs on demand, without the possibility of adjusting the respiratory rate. In this mode, mandatory IPAP and EPAP levels are preset, and in some devices, other variables can also be adjusted, such as rise and fall times, inspiratory time, etc. This mode is rarely used in home ventilation.

#### S/T or E/T mode

A back-up respiratory rate is preset in case the patient does not reach the predetermined respiratory rate within a time window of 60 seconds, that is, if the patient does not breathe spontaneously, a mandatory breath will be released.

#### T mode

A respiratory rate is preset, and ventilation occurs according to this parameter, regardless of patient effort. It is not recommended for patients in whom the respiratory drive is preserved owing to the possibility of discomfort and asynchrony.

#### PAC mode

It is a time-cycled, pressure-limited ventilator mode. The device is triggered by the patient's inspiration or after a preset period of time if the patient's spontaneous respiratory rate falls below the back-up respiratory rate. The inspiratory time should be preset, so expiration occurs after this period of time. Inspiration may be either machine or patient triggered, but all breaths are cycled by the ventilator.

#### SIMV mode

SIMV stands for “synchronized intermittent mandatory ventilation”. A patient inspiratory effort generates an additional device cycle, or a pressure is generated at a predetermined flow rate (time-cycled, pressure-limited SIMV mode), or inflated at a predetermined volume (volume-cycled SIMV modes). The next cycle is automatically delayed. As in the S/T mode, a minimum respiratory rate is preset.

#### VAC mode

The tidal volume or minute volume is delivered at set intervals of time despite changes in airway resistance or lung compliance. Failure to ensure volume may occur in the presence of leaks. There is a risk that high pressures will be required to deliver the predetermined volume.

#### Volume-targeted pressure-controlled modes (AVAPS® or iVAPS®)

The target tidal volume or minute volume can be adjusted in some devices. In this mode, the ventilator delivers a target volume by varying the pressure within a maximum and minimum IPAP delta.

### Types of interfaces used in HMV

**Figure 1:**
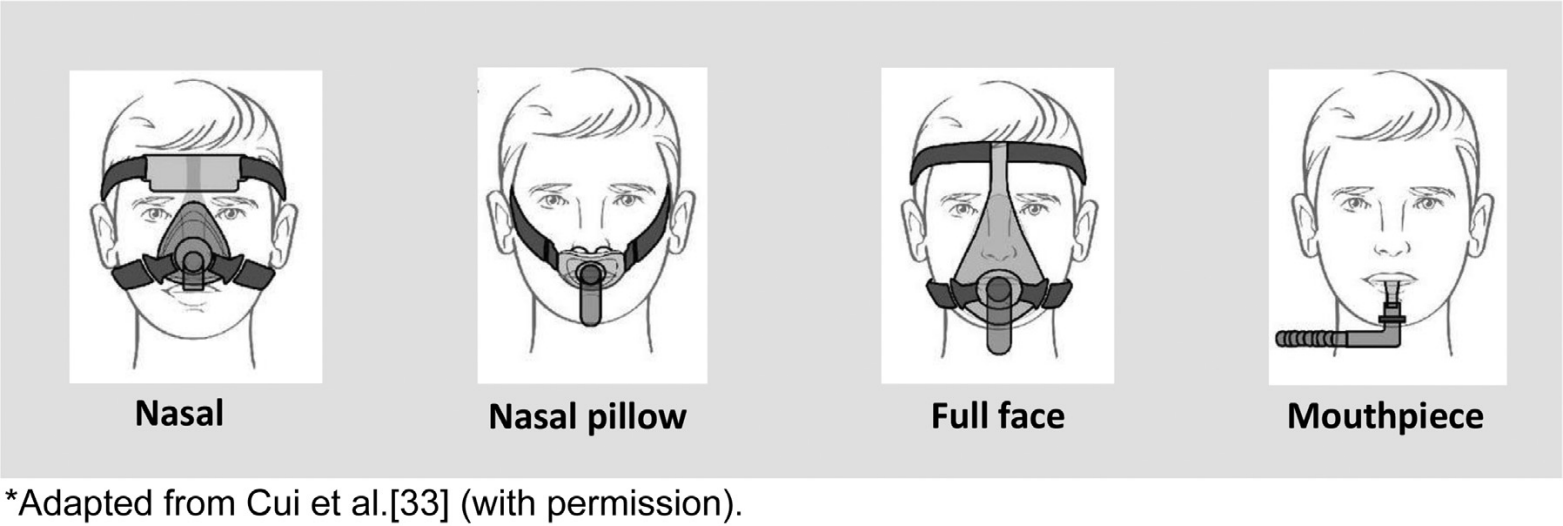
Types of interfaces used in HMV. *Adapted from Cui et al.^33^ (with permission).

In the noninvasive modality, HMV is usually delivered by means of a single-limb circuit using vented masks with intentional leak, namely, masks that have holes in their structure to allow expiration (Fig. 1).^33^ In some cases, a double-limb circuit or a single-limb circuit with an expiratory valve may be required, in which case non-vented masks will be used.

### Types of circuit

Two types of circuits are most commonly used in home ventilators.^32^

#### Single-limb ventilator circuit

Inspiration and expiration occur through a single line. Expiration will occur through the intentional leak on the interface when ventilation is noninvasive. In invasive ventilation (via tracheostomy), expiration will occur through an expiratory valve that is specific to each ventilator. In these cases, the inclusion of an expiratory valve in the circuit is mandatory, or alternatively a single-limb circuit with an exhalation valve can be used.

#### Double-limb ventilator circuit

An active exhalation system is used. In NIV, a non-vented mask type is required, otherwise the exhalation holes on the mask must be occluded.

### Humidification system, filters, expiratory valve, and supplemental oxygen therapy^1^^,^^5^^,^^34^^,^^35^

#### Humidification systems and filters

The goal of ventilatory support is to be as physiological as possible. Therefore, a heated and humidified system aiming to maintain the natural balance of heat and humidity in the airways is desirable. Two forms are currently used: (1) Heated humidification and (2) Humidification through a Heat and Moisture Exchanger (HME) device.

#### Heated humidification

This method uses an external power supply with a water reservoir. This type of heating requires rigorous cleaning of the reservoir to prevent the proliferation of microorganisms.

#### HME filters

These devices filter and recycle the patient's own heat and moisture.

### Barrier filter

For patients using second-hand equipment (e.g., obtained via rental, home care, or public ventilation programs), a barrier filter capable of effectively filtering airborne particles (both viruses and bacteria) is recommended. It should be replaced periodically according to the manufacturer's instructions.

### Expiratory valve

In NIV, vented interfaces that allow intentional leaks through the passive exhalation port are used to avoid Carbon Dioxide (CO_2_) rebreathing. As previously mentioned, the inclusion of an expiratory valve in the circuit is mandatory for patients with a tracheostomy, or alternatively a single-limb circuit with an exhalation valve can be used.

### Supplemental oxygen therapy

When required in home ventilators, Oxygen (O_2_) supplementation should be provided through the oxygen inlet usually located at the rear of the machine. In bilevel machines, O_2_ supplementation should be provided through a connector placed next to the device outflow port (away from the mask). It should be noted that the FiO_2_ delivered to the patient will depend on the O_2_ flow, connection position of the O_2_ source in the circuit, degree of leakage in the device's circuit, type of interface used, IPAP and EPAP levels offered, and the patient's own ventilatory biomechanics.

### Criteria for selection of equipment for HMV^12^^,^^13^^,^^35^

**Figure 2:**
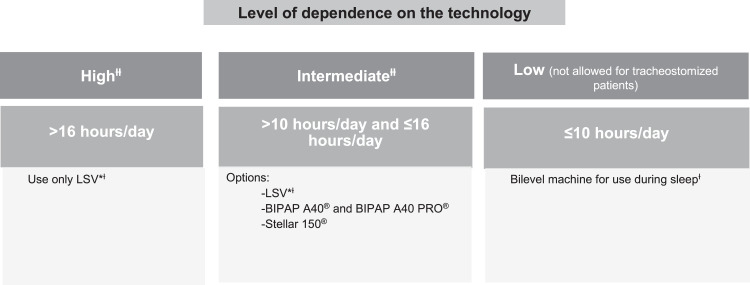
Choice of equipment for noninvasive and invasive HMV in adults. * LSV: life support ventilator. ^Ɨ^ See Table 3. ^ƗƗ^ For tracheostomized patients (invasive HMV), only “Intermediate” and “High” level of dependence on the technology should be considered).

**Figure 3:**
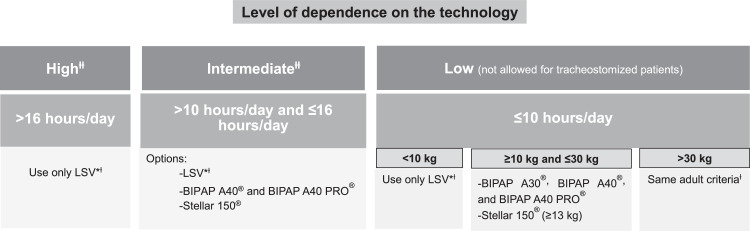
Choice of equipment for noninvasive and invasive HMV in children. * LSV, Life Support Ventilator. ^Ɨ^See Table 3. ^ƗƗ^ For tracheostomized patients (invasive HMV), only “Intermediate” and “High” level of dependence on the technology should be considered.

The decision about the type of equipment to be used at home should take into account the level of dependence, that is, the number of hours per day that ventilation will be required, and the presence of a tracheostomy. In pediatric patients, it is also necessary to take body weight into account. Figure 2, Figure 3 show the selection of equipment for adult and pediatric patients, respectively. A comparison of devices for home ventilatory support available in the Brazilian market is shown in Table 3.

**Table 3:** Comparison of devices for home ventilatory support available in the Brazilian Market.^a^

Low dependence on equipment (≤ 8 to 10 hours/day)	Intermediate dependence on equipment (≤ 16 hours/day and/or in tracheostomized patients)	High dependence on equipment or life support (> 16 hours/day and/or body weight < 10 kg and/or in tracheostomized patients)
AirCurve 10 ST® ‒ ResMed. Use in patients with body weight ≥ 30 kg; IPAP maximum of 30 cmH_2_O.	Stellar 100® and Stellar 150® ‒ ResMed. Use in patients with body weight ≥ 13 kg; IPAP maximum of 40 cmH_2_O.	Astral 100® and 150® ‒ ResMed. Use in patients with body weight ≥ 5 kg.
Dream ST AVAPS® ‒ Philips-Respironics. Use in patients with body weight ≥ 18 kg; IPAP maximum of 30 cmH_2_O.	BIPAP A40® ‒ Philips-Respironics. Use in patients with body weight ≥ 10 kg; IPAP maximum of 40 cmH_2_O.	Trilogy 100® ‒ Philips. Use in patients with body weight ≥ 5 kg.
BIPAP A30® - Philips-Respironics. Use in patients with body weight ≥ 10 kg; IPAP maximum of 30 cmH_2_O.	BIPAP A40 PRO® ‒ Philips-Respironics. Use in patients with body weight ≥ 10 kg; IPAP maximum of 40 cmH_2_O.	Puritan ‒ Bennett 560® ‒ Coviden. Use in patients with body weight ≥ 5 kg.
BIPAP Synchrony AVAPS® ‒ Philips-Respironics. Use in patients with body weight ≥ 18 kg; IPAP maximum of 30 cmH_2_O.		Monnal T50® ‒ AirLiquide. Use in patients with body weight ≥ 5 kg.
Breath Care PAP® model YH-30 ‒ Yuwell. Use in patients with body weight ≥ 30 kg; IPAP maximum of 30 cmH_2_O.		Trilogy Evo® ‒ Philips Use in patients with body weight ≥ 2.5 kg.
DreamStar Duo ST® ‒ Sefam. Use in patients with body weight ≥ 30 kg; IPAP maximum of 25 cmH_2_O.		Prisma VENT 50® – Lowenstein. Use in patients with body weight ≥ 10 kg.
Prisma Vent 30® – Lowenstein. Use in patients with body weight ≥ 30 kg; IPAP maximum of 30 cm H_2_O.		EO-150 EOVE® ‒ Air Liquide Medical System. Use in patients with body weight ≥ 3.5 kg.
BPAP Resmart ST GII T-30T® ‒ BMC. Use in patients with body weight ≥ 30 kg; IPAP maximum of 30 cmH_2_O.		TIVAN 50® – Lowenstein. Use in patients with body weight ≥ 10 kg.
Bilevel SleepCube S/T® ‒ Devilbiss. Use in patients with body weight ≥ 25 kg; IPAP maximum of 30 cmH_2_O.		
BiPAP Series 7 - ST730W® ‒ Hypnus. Use in patients with body weight ≥ 30 kg; IPAP maximum of 30 cm H_2_O.		

a For more information, please consult the equipment user manual.

### Minimum requirements for the implementation of home ventilation^7^^,^^13^^,^^34^

A wide variety of unique and important social and medical issues will need to be considered when planning for the care of each individual patient. If the care is family and patient centered, recognition of patient and family preferences, social services availability, barriers to communication, or medical provision will be necessary.^36^

HMV requires monitoring by a team trained to provide care at the household level. The clinical conditions of the person to be assisted, the family support (with the definition of the caregivers) and the home structure (including home electrification conditions, clean and well-ventilated bedroom) should be carefully evaluated aiming at patient and equipment safety and treatment effectiveness.

An individualized approach to HMV settings may benefit patients with chronic respiratory failure and sleep-disordered breathing when indicated. The clinician's role is to add evaluation at the bedside for shared decision-making with patients and families, including respect for patient preferences and treatment goals, considerations of quality of life, and appropriate use of available resources in decision-making.^24^

To provide home ventilation, the following are required:^37^

Equipment suitable for home ventilatory support;
Interface (if NIV): mask, mouthpiece;
Ventilator circuit and back-up circuit;
Heated humidifier;
External battery;
Bag-valve-mask manual resuscitator (“Ambu” bag)⁎⁎⁎⁎;
Desktop pulse oximeter with a plethysmographic waveform display;
Portable airway suction device.

The equipment for ventilatory support is portable, powered by electricity, and dual voltage. Only intermediate and life support devices have an internal battery, and they should remain connected to electricity at all times to prevent battery discharge. In some situations, an Uninterruptible Power Supply (UPS) unit is coupled to the equipment in use and the following precautions should be taken:

The equipment electric charge and uninterrupted connection to an electrical power source should be checked daily to ensure that it is working properly (power strips or extension cords should not be used).

Only the equipment and the heated base should be connected to the UPS unit. No other electronic equipment should be connected to this device.

The UPS unit should not be transported due to the risk of falling and burning.

Household resources and conditions should be evaluated and monitored regularly, such as safe water and electricity supply, adequate electrical circuits for the ventilator, landline, or cell phone for contacting the rescue team, and adequate storage space for the equipment. Patients who use medical devices that depend on electricity to operate should register with the energy supplier in their city to be notified in advance of any planned power outage.

#### Contraindications to the use of bilevel ventilators^1^^,^^13^^,^^17^

• Patient with a tracheostomy (except for BiPAP A40®, BiPAP A40 PRO® and Stellar 150®);
• More than 16 hours of ventilation required per day;
• Presence of abundant pulmonary secretions and/or impossibility to adequately remove the secretions;
• Altered mental status with impaired ability to understand and cooperate;
• Presence of clinical instability (hemodynamic and/or respiratory) within 48 hours of hospital discharge;
• Absence of family and social support to implement safe HMV.

#### Contraindications to the use of life support ventilators^1^^,^^13^^,^^17^

• Presence of abundant pulmonary secretions and/or impossibility to adequately remove secretions (patients on NIV);
• Altered mental status with impaired ability to understand and cooperate (patients on NIV);
• Presence of clinical instability (hemodynamic and/or respiratory) within 48 hours of hospital discharge;
• Absence of family and social support to implement safe HMV.
• Contraindications should be considered within the clinical context of each patient, assessing the risks and benefits of each situation in an individualized and personalized way. Therefore, in some cases, the aforementioned contraindications may be considered relative. At the physician's discretion, other medical conditions that might prevent the use of positive airway pressure should be considered before ventilation is indicated.

#### Required conditions for the implementation of home ventilatory support^14^^,^^32^^,^^37^

• Exclusive and individual outlets for each device used in patient care;
• Physical environment free of objects/furniture that might cause falls, disturb walking or that might fall on the patient;
• Well-ventilated environment (if necessary, a fan or air conditioning can be used), with good lighting and protection against insects and other venomous animals (window screens, etc.). Washable roof lining, floor, and painting;
• Accessibility around the home for emergency medical service access;
• Contingency plans for complications (accidental decannulation, power outage, technical problems with the ventilator), and periodic inspections with family members and/or caregivers.

### HMV equipment care^32^^,^^35^^,^^36^

#### Equipment

Wipe the outside of the device with a dry cloth or, if necessary, with a moist cloth. Never spray liquid directly on the equipment or use abrasive cleaners.

Keep the fan on a flat and stable surface to prevent it from falling.

Place the equipment at the bed head level or in a lower position.

Do not leave the equipment close to electromagnetic devices, such as defibrillators, cell phones, and others.

Do not smoke in the room where the ventilatory support equipment and/or the oxygen cylinder is installed.

Do not expose the equipment to direct sunlight and do not cover it with any material that prevents cooling air passages.

Do not place liquids or food near the equipment and its accessories.

The electrical installation must support the electrical power indicated in the equipment user manual.

#### Interface

Wash it at least once a week with water and mild liquid soap.

Do not use products containing alcohol, vinegar, or hydrogen peroxide, since these products dry out the material and reduce the lifetime of the mask.

Allow the mask to dry in a well-ventilated location out of direct sunlight.

#### Heated humidifier

Change the humidifier water daily.

Use only double-distilled water or filtered and boiled water (after cooling). Never use saline or any other product in the humidifier reservoir.

Wash it at least once a week with water and a small amount of mild liquid soap.

In equipment without an integrated heated base, the humidifier cannot be placed in a position above the equipment. If water condensation occurs, this measure will prevent liquid from entering the circuit and interface or the tracheal tube, avoiding damage to the equipment due to accidental water entry.

### Regulatory affairs of home ventilatory support

#### Brazilian Health Regulatory Agency (ANVISA)

According to the manual for the regularization of medical devices at ANVISA, published in October 2017, ventilators are classified as active medical devices and their lifetime is defined according to treatment duration: transient, short term, and long term (regular use). ANVISA determines that the equipment should be used strictly according to the manufacturer's instructions. For detailed information, please refer to the “Manual for regularization of medical devices at ANVISA”, version 1.5, of October 3, 2017, available at www.anvisa.gov.br. ^38^

#### International Organization for Standardization (ISO)

ISO, in its standards ISO 10651-2:2004 (available at https://www.iso.org/standard/35973.html)^39^ and ISO 80601-2-72:2015 (available at https://www.iso.org/standard/61389.html),^40^ also defines and classifies the types of ventilators and their lifetime according to treatment duration. As in the ANVISA manual, ISO standards establish the need to use the ventilator and its accessories according to the manufacturer's instructions to ensure proper use of each equipment model. The aforementioned documents address and standardize clinical situations in which the use of a life support ventilator is mandatory.

## Conflicts of interest

The authors declare no conflicts of interest.

## Footnotes

⁎⁎⁎⁎ Indicated for use in case of ventilator failure, power outage, or in any other emergency situation. The bag-valve-mask device should be checked periodically to ensure safe conditions of use. It should be kept in an easily accessible place, clean and protected from light and heat.
